# Functional Imaging in Diagnostic of Orthopedic Implant-Associated Infections

**DOI:** 10.3390/diagnostics3040356

**Published:** 2013-10-21

**Authors:** Inga Potapova

**Affiliations:** AO Foundation, Clavadelerstrasse 8, 7270 Davos, Switzerland; E-Mail: inga.potapova@aofoundation.org; Tel.: +41-81-414-2445; Fax: +41-81-414-2288

**Keywords:** orthopedic implant associated infections, diagnostic imaging

## Abstract

Surgeries’ sterile conditions and perioperative antibiotic therapies decrease implant associated infections rates significantly. However, up to 10% of orthopedic devices still fail due to infections. An implant infection generates a high socio-economic burden. An early diagnosis of an infection would significantly improve patients’ outcomes. There are numerous clinical tests to diagnose infections. The “Gold Standard” is a microbiological culture, which requires an invasive sampling and lasts up to several weeks. None of the existing tests in clinics alone is sufficient for a conclusive diagnosis of an infection. Meanwhile, there are functional imaging modalities, which hold the promise of a non-invasive, quick, and specific infection diagnostic. This review focuses on orthopedic implant-associated infections, their pathogenicity, diagnosis and functional imaging.

## 1. Implant’s Failures

Implant-associated aseptic failures and infections are the health threatening complications which orthopedic devices may develop during their life time. Aseptic loosening and infection are the main reasons of an implant failure. These pathologies are causatively different, though similar in clinical presentation. Thus, it has been found that many previously assigned aseptically loosened implants indeed failed due to a low grade infection which was not recognized [1,2].

Aseptic failures may happen in response to the following: (1) wear implant debris may cause inflammation mediated osteoclastic bone absorption (the “arthroplasty effect”), as well as distant dissemination of debris; (2) inappropriate mechanical load; (3) fatigue failure at bone-implant interfaces; (4) implant micromotion; (5) synovial fluid hydrodynamic pressure [3]; allergy (hypersensitivity) to metal [2].

An infection incidence is based on an invasion of peri-prosthetics by microorganisms. Orthopedic devices undergo a physiological change after implantation. After the implant insertion, body fluids immediately coat its surface with conditioning film made of water, lipids, complement, inorganic salts and extracellular matrix (ECM) molecules such as albumin, fibronectin, fibrinogen, vitronectin, elastin, collagen, *etc.* [4,5]. The earliest and clinically principal step is the competition between host tissue cell integration and bacterial adhesion to the foreign surface of the inserted device [6]. Infections become established when the dose of bacteria with its inherent virulence overcomes host defenses and colonize the implant surface forming biofilms.

Inflammation is the general term for both: (1) the response of an organism to an exogenous pathogen, which is called an infection; and (2) the response to tissue injury, called an aseptic inflammation [7,8]. Visual signs of infectious and aseptic inflammation are very similar but their clinical management is different; therefore it is necessary to be able to distinguish between aseptic and infected implant. An aseptic implant must be removed and exchanged. An infected implant is managed by a prolonged and pathogen specific antibiotics therapy. There are one- or two-stage revisions with application of systemic and local antibiotics, which are used to manage infected implants. Only in rare cases of low and controllable infections, an antibiotics therapy without re-implantation could be applied, for instance DAIR—Debridement, Antibiotics and Implant Retention [9].

## 2. Pathogenicity of Infections

Implant-associated infections still occur despite hospitals’ sterile conditions and antibiotics therapies, which have significantly decreased rates of infection. Thus, Prosthetic Joint Infection (PJI) has 1%–9% infection rates [9,10]. Fracture Fixation Infection (FFI) has only 1%–2% infection rates for closed fractures but up to 30% for open fractures [11]. Moreover, infections after revision surgeries re-occur quite often [12,13]. 

Infections may be classified by the onset of symptoms after implantation (early, delayed, and late) and route of infection (exogenous, contiguous, hematogenous), see Table 1. It is important to note, that implanted devices remain susceptible to blood transported bacteria during their entire life and some perioperative infections may have a latency period over two years [10]. Prevalently, early infections are caused by highly virulent (pathogenic) microbes; delayed infections are caused by low virulent ones (such as commensals); late infections are caused by remote infections of pathogenic character. The bacteria tend to infect an implant during trauma or implant surgery (perioperative infections). Principal microorganisms causing infections are Gram Positive *Staphylococcus aureus* (*S. aureus*) and *Staphylococcus epidermidis* (*S. epi*). Gram negative *Escherichia coli* (*E. coli*), *Pseudomonas aeruginosa* (*P. aeruginosa*) and *Propionbacterium acnes* (*P. acnes*) were found on infected implants as well, but less frequently. Gram positive Strepto- and Enterococci appear preferentially during a later infection phase, predominantly by hemotogenous seeding.

**Table 1:** Classification of infections adapted from [11,14,15].

Classification	Infection organism	Prosthetic Joint	Fracture Fixation
**Onset of symptoms after implantation**			
Early	*Staphylococcus aureus (S. aureus)* *Staphylococcus coagulase-negative* *Aerobic Gram-negative bacteria*	<3 months	<2 weeks
		acquired during implant surgery or in the following 2 to 4 days and caused by highly virulent organisms	acquired during trauma or implant surgery, caused by highly virulent organisms
Delayed	*Staphylococcus coagulase-negative* *Skin bacteria: Staphylococcus epidermidis (S. epi), Propionibacterium acnes (P. acnes)*	3–24 months	2–10 weeks
		acquired during implant surgery and caused by less virulent organisms	acquired during trauma or implant surgery and caused by low virulence organisms; or caused by hematogenous seeding from remote infections
Late	*S. aureus; Methicillin resistant S.aureus (MRSA)* *Staphylococcus coagulase-negative* *Skin bacteria (S. epi); Anaerobes (P. acnes); Escherichia coli (E. coli)*	>24 months	>10 weeks
		Predominantly caused by hematogenous seeding from remote infections	
**Route of infection**			
Perioperative		Inoculation of microorganisms into the surgical wound during surgery or immediately thereafter	
Contiguous		Spread from an adjacent focus of infection: penetrating trauma, preexisting osteomyelitis, skin and soft tissue lesions	Wound contamination due to penetrating trauma (open fractures) or from an adjacent focus of infection (skin and soft-tissue lesions)
Hematogenous		Microbial spread through blood or lymph from a distant focus of infection: skin, lung, urinary tract	
**Microorganisms**		**Infection frequency (%)**	
Staphylococcus coagulase-negative, *S. epi*		37	22
Staphylococcus coagulase-positive, *S. aureus*		18	30
Streptococci		10	1
Enterococci		5	3
Gram-negative bacilli; *E.coli*, *P. aeruginosa*		5	10
Anaerobes; *P. acnes*		3	5
Polymicrobial		11	27
Unknown		11	2

## 3. Bacterial Ways to Cause Implant-Associated Infections

Bacteria may become resistant to applied antibiotics [16], hide intracellularly [1] and form biofilms [17]. The most common form of bacteria persistence is to develop a resistance to conventional antibiotics. Thus the resistant bacteria do not respond to the respective elimination therapies. Besides this, bacteria readily reside intracellularly and in biofilms. 

A formation of small colony versions (SCV) is bacteria’s unique way to avoid clearance. SCV bacteria do not respond to an immune or antibiotics attack and readily reside intracellularly (found in osteoblasts) and in biofilms. It has been found that this form of bacteria is responsible for the high recurrence of musculoskeletal infection after revision surgeries [1].

Conventional implant-associated infections are connected to the formation of a biofilm. Bacteria colonize prosthetic materials forming a biofilm: “a community of microbes embedded in an organic polymer matrix, adhering to a surface” [4]. Hydrophobic materials like Teflon and other plastics are more susceptible to bacteria colonization then hydrophilic glasses and metals. Rough surfaces favor biofilm formation more than smooth ones. This is due to the reduction of shear stress and increased surface area [18,19]. 

**Figure 1:**
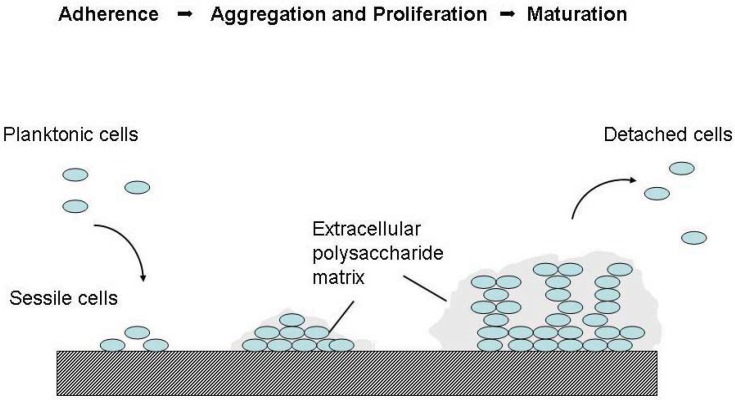
Biofilm formation (reprinted from [20] with permission from Elsevier^®^).

The biofilm formation starts from a bacteria adherence to an implant, mediated by the interaction between host matrix-binding proteins and bacterial adhesins on the surface of the implant. Subsequently, bacteria aggregate and proliferate producing extracellular polysaccharide, proteins and eDNA (extracellular DNA) matrix on the prosthetic surface. Encased in the matrix, bacteria mature into a slimy layer in the form of a biofilm, see Figure 1. 

Biofilms grow slowly (over a period of several hours to several days) and resist cellular and immune responses. In a matured biofilm, bacteria communicate using “quorum sensing” small molecules. Matured biofilms are composed of a sophisticated network of microcolonies, channels and voids for efficient nutrient supply and prolonged bacteria survival [17,21]. Sessile (biofilm-associated) bacteria may detach as microcolonies as well as planktonic (single) bacteria and seed on virgin surfaces promoting the spread of infection. 

Most antimicrobials fail to penetrate the biofilm matrix. Moreover, bacteria within a biofilm are very robust against common anti-bacterial means. Thus, the availability of antibiotics is limited by the biofilm-related pharmacokinetic requirements. Rifampicin, Vancomycin, Gentamicin, Teicoplanin, Linezolid, Cafazolin, which are used alone or in combination, are the clinical options to prevent a prosthetic biofilm development [22]. These antibiotics are used in a prolonged period treatment and at elevated amounts. In clinical practice, the systemic antibiotics therapy (injection of soluble antibiotics) is mandatory to kill the planktonic bacteria in perioperative space. Systemic antibiotics are also mandatory at least for a couple of weeks postoperatively. Nowadays, in addition to the conventional systemic antibiotics therapy, there is a possibility to use commercially available antibiotics-coated implants (local prophylaxis therapy) to avoid an adhesion of bacteria on the implants [23]. Local antibiotics are also used in a two stage revision surgery: after removal of a failed implant and debridement and before a new implant is inserted, antibiotics-loaded beads are often inserted to clear the peri-implant space of any pathogens.

There are many innovative approaches to fight implant-associated infections based on disruption of biofilms. Some promising strategies are based on applying enzymatic anti-biofilm agents; gene therapies to interfere with biofilm production; quorum sensing inhibitors; photodynamic activation and subsequent exposure to visible light; biofilm preventive molecules [24]. Yet these new strategies have not yet been clinically validated.

## 4. Infection Diagnosis

Implant-associated intracellular and biofilm related bacteria are masked from any conventional diagnostics assessment. Moreover, differentiation of infection conditions from aseptic inflammation is difficult; both conditions are very similar clinically and histopathologically. Clinical signs, symptoms, laboratory tests and radiography of these pathologies are often non-distinguishable. Thus, in the postoperative period, signs and symptoms that are associated with infection are masked by normal postoperative changes. A wide range of tests is used to detect an infection [25]. There are clinical examinations, imaging tests, serology, microbiology, molecular techniques, sonication and calorimetry, which are classified as perioperative or intraoperative tests. Appendix 1 “Infection Diagnostics Tests” (Supplementary Information) gives the detailed description of the tests and their limitations in clinical practice.

**Figure 2:**
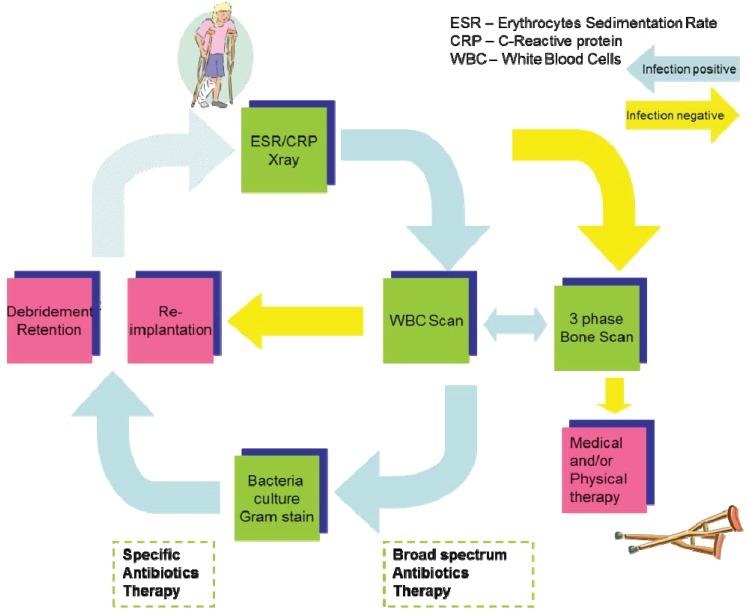
Flow chart of infection diagnosis in clinics.

When a patient claims an implant-associated discomfort, there is a general run of tests to diagnose and apply an appropriate treatment: see Figure 2, Flow Chart of Infection Diagnosis in Clinics. Patient conditions and clinical presentation (such as pain, swelling, pus formation, redness *etc.*) are the first hints of an infection. At this step clinicians perform ESR/CRP blood tests and X-rays. ESR-erythrocyte sedimentation rate, CRP-C-reactive protein and WBC (White Blood Cells) are very sensitive parameters of trauma-related inflammatory processes. Elevated read-outs of these parameters and/or abnormalities seen on X-rays are the basics to confirm a suspicion of an infection. The next step to confirm an infection is WBC (White Blood Cells) and/or combined WBC/bone scans. Autologous, radiolabeled WBC and MDP (Methylene DiPhosphate) as leukocytes and bone tracers, respectively, are injected into the patients’ blood stream where they localize to inflamed sites and can be visualized by Scintigraphy, SPECT or PET. If there is an infection positive read-out, clinicians start with broad spectrum antibiotics therapy. Meanwhile tissue and swab probes from peri-prosthetics (taken invasively) are subjected to microbiological tests. A bacteriological (microbiological) culture may last several weeks. The grown bacteria (or fungi) are the “Gold Standard” in diagnosis of an infection. The identification of the bacteria profiles on the basis of the bacteriological cultures determines specific antibiotics and surgery strategies to treat patients.

## 5. Diagnostic Imaging Modalities

Imaging offers the prospect of easy, sensitive, non-invasive, specific, and patient friendly screening of infection. Imaging techniques have wide range of wavelengths, different mechanisms, applicability and limitations; see Appendix 2 in Supplementary Information, Infection Imaging Techniques. Imaging techniques are capable to visualize an anatomical and functional localization of an implant-related infection and emerge to play an important role in the clinical diagnosis of infection.

Morphological or anatomical imaging include X-ray plane radiograms (X-ray) and 3-dimentional techniques, such as computed tomography (CT), ultrasonography (USG) and magnetic resonance imaging (MRI), which give the anatomical resolution. Anatomic imaging modalities provide high-quality details and are widely available. However, conventional CT and MRI are disturbed by the presence of metallic implants and are not sensitive to early infection stages, where obvious abnormalities do not appear. USG cannot visualize bones and is limited to soft tissue abnormalities. Overall, conventionally, the early postsurgical diagnosis using USG, CT and MRI cannot consistently separate infection from normal postoperative changes [26]. 

Functional imaging enables visualization of pathologic processes. Optical functional imaging of infections is represented by Fluorescence modality (see Appendix 2 in Supplementary Information) and limited to *ex vivo* and *in vivo* preclinical models due to limitations in light penetration depth. Recently optical imaging found its application in intraoperative imaging [27]. Functional imaging also can be realized with CT and MRI using appropriate contrast agents (*i.e.*, nanoparticles) conjugated to biologically active probes. However, mostly due to artifacts of metal implants, CT and MRI were not employed widely in functional imaging.

Functional imaging using radioactive isotopes is called Nuclear Medicine. It is widely used in clinical infection diagnostic. Nuclear images are obtained via detection of radiopharmaceuticals. A radiopharmaceutical is a radionuclide compound in which a radioisotope (radionuclei) is conjugated to a biologically active molecule (probe). Radiopharmaceuticals trace physiological changes when administrated to a patient. Nuclear medicine traces physicochemical changes and may provide a functional evaluation of infection. The infection-related physicochemical changes include increased blood supply and vascular permeability, enhanced transdulation of plasma proteins and influx of leukocytes in response to infection or inflammation. Mediators of these changes can be labeled and traced *in vivo*.

Plane Scintigraphy, SPECT (single photon emission computed tomography) and PET (positron emission tomography) are the imaging modalities of Nuclear Medicine [28]. In plane Scintigraphy and SPECT, radioactive isotopes emit gamma-rays and isotopes’ distribution in a patient is detected. PET tracers emit positrons, which detection allows improved spatial resolution. SPECT and PET enable three dimensional imaging. However, PET technique is quite expensive because of costs for the production of positron emitters and sophisticated detection cameras. 

Combined with CT hybrid SPECT/CT and PET/CT advanced modalities give fair anatomical localization of infection and improved accuracy in infection detection [29,30,31,32]. These powerful dual-modality techniques integrate both, physiology and anatomy, thus far allowing improved imaging of implant associated infection-inflammation puzzles [32,33,34,35,36,37,38,39].

### 5.1. Radioisotopes and Radiolabeling

Performance of radioisotopes is very important for the imaging quality and human safety. Physical description of common isotopes used in Scintigraphy, SPECT and PET is given in Table 2. 

**Table 2:** Common radioisotopes used in infection imaging.

Tracer	Emission Max keV	Spatial Resolution	Half Life hr	Postinjection Imaging hr	Radioactive dose
**Gamma emitters/SPECT**					
111 In	173; 247	Low	67	18–24	High
99m Tc	140	High	6	>3	Low
67 Ga	93; 184; 296	Low	78	24–72	High
**Positron emitters/PET**					
68 Ga	1.9 × 1000	High	1.1	1.1–1.5	Low
18 F	6.4 × 100	High	1.8	0.5–1.5	Low

*Gamma-emitting radioisotopes*. 67 Ga has been widely used in clinical practice in earlier days. Albeit this isotope has poor physical characteristics for gamma imaging: it has a long half-life and decays in a broad range of gamma-ray emissions. 111 In has also the long half-life time, but high *in vivo* stability allowing the acquisition of late images. However the emission spectrum of 111-In is also suboptimal for acquiring by gamma cameras. The concentration 111 In in infection foci has to be higher than for other gamma-emitting isotopes, which might be the source of a higher radioactive burden to a patient. Additionally, 111 In harvesting and labeling procedures are time consuming and complex [40]. 99m Tc has optimal physical characteristics for gamma-camera imaging, easier handling, and produces well-resolved images [32], therefore it is widely preferred to “old” 67 Ga and 111 In. 

*Positron-emitting radioisotopes.* 68 Ga [41] and 18 F [42] along with other positron emitters such as 124 I [43] and 64 Cu [44,45] are the isotopes, which are used in PET, PET/CT, PET/MRI. The expensive production and handling of PET probes together with high costs of PET cameras are the serious obstacles for the PET technology progress. However PET Diagnostic is getting popular due the best imaging performance.

There are indirect and direct methods to label infection probes with radioisotopes. In an indirect conjugation, there is a chelating molecule between an isotope and an infection probe. Oxine, HYNIC (6-Hydrazinopyridine-3-Carboxylic Acid) and HMPAO (HexaMethylPropylene Amine Oxime) are chemically well defined chelating agents and frequently used for indirect conjugations. In a direct conjugation, an infection probe is chemically conjugated to a radioisotope via available free amine, carboxyl or sulfide groups of probes. For instance the direct labeling of UBI29-41 [46,47,48,49,50,51,52] with 99m Tc results in a dimeric peptide-Tc complex via UBI’s amine groups of Arg and Lys [53].

The major limitation of all radiolabeling methods is the careful purification to remove any traces of unconjugated probes [54]. Moreover, conjugations may disturb activity and have an impact on the biological performance and pharmacokinetics of the probes. Thus, some conjugates have shown altered behavior as it was reported for 99m Tc-ciprofloxacin [55]. In particular, for 99m Tc-ciprofloxacin, the radiolabeled antibiotic, is not recognized by the bacteria efflux pumps and, in contrast to ciprofloxacin alone, and it accumulates inside bacteria [48]. Another example of the radiopharmaceutical misbehavior is dissociation upon *in vivo* registration. Tc-HMPAO is quickly eluted from labeled WBC (white blood cells), released into the blood and excreted through the kidneys and intestine [40].

### 5.2. Infection Probes

The most explored infection targets are WBCs, bacterial surface and DNA [49]. Localization of WBCs (in particular, neutrophils) to bacterial invasion sites occurs as the result of the host innate immunity response. A negatively charged bacterial surface attracts positively charged antimicrobial peptides (AMPs). Bacterial DNA is targeted by antibiotics molecules. Thus, conventional infection probes are labeled WBCs, AMPs and antibiotics, respectively. Poly- and monoclonal antibodies are also used to target bacteria and WBCs [7,51,52,54,56]. The wide range of probes used in preclinical models and clinical studies is summarized in Appendix 3 in Supplementary Information, Clinically Relevant Radiolabeled Probes in Implant-Related Infection Imaging. Many of probes are claimed to be very efficient; some have been already commercialized, but further clinical trials have not proved their absolute excellence for infection diagnostic. 

The recent trend to discriminate between infection and sterile inflammation is to trace bacteria or bacteria produced factors [51,54], which are directly related to bacterial infection, see Appendix 3 in Supplementary Information.

Here we would like to comment on some infection probes in the context of their applicability in clinical diagnostic of implant-related orthopedic infections. Thus, 99m Tc-Fanolesomab (anti-CD15), monoclonal antibody against neutrophils’ receptors, was withdrawn from USA market due to deaths occurred after the probe’s administrations. Although the deaths may be not linked to Fanolesomab the future of this probe for human use is unknown [57]. The most explored radiolabeled antibiotics, 99m Tc-ciprofloxacin (Infecton^®^) was intensively debated in the literature due to its drawbacks in practical use [48,58,59,60,61,62,63,64,65,66,67,68,69,70]. Up-to-date, the synthetic antimicrobial peptide, 99m Tc-UBI 29–41 (fragment of Ubiquicidin) is the most promising probe [71,72,73,74,75,76,77,78]. This probe showed excellent sensitivity, specificity and accuracy to infections (95%) in clinical trials [79]. 

Combined 99m Tc-MDP/111 In-WBCs and combined 99m Tc-colloid bone marrow/111 In WBCs become the “method of choice” [80,81] for infection imaging in clinics. A traumatized bone is haematopoietically active and takes up bone/bone marrow probes (MDP or sulfur colloid) as well as WBC. Using both radiolabeled marrow-bone probes and WBC allows distinguishing between infection and trauma-associated inflammation. The bone and marrow probes’ uptake is infection non-specific, while the WBCs’ uptake has both infection non-specific and specific components. Thus, there is the non-congruent (mismatching) bone and WBC uptake in case of septic and the congruent (matching) uptake in an aseptic inflammation. However, even this “method of choice” has limitations, such as blood handling with risk of HIV and hepatitis infections; altered function of WBCs in patients under therapy, *etc.* (see Appendix 3 in Supplementary Information). 

18F-FDG (Fluorodeoxyglucose), the most used PET probe, is taken up by cells with enhanced metabolism, and enables visualization of hyperglycolic inflammatory cells (leukocytes, macrophages, and other immunologically active cells) during infection. 18F-FDG PET was considered as an attractive alternative to WBCs-bone marrow scintigraphy because it requires only one injection of 18F-FDG, and it is not affected by antibiotics therapy. Moreover FDG uptake does not rely on WBC migration [29]. FDG PET has the superior resolution and advantage of blood-free quick [82] processing but its ability to specify precisely infection is debatable [29,31,38,80,83,84,85,86,87,88]. The concerns about FDG PET are mostly due to the FDG non-specific uptake localized along the interface between bones and implants. Thus, in patients with an inflammation due to peri-prosthetic aseptic foreign-body (metal or plastic) reactions, FDG-PET specificity to infection may be low. One more complication of FDG-PET is an interpretation of images and fair diagnostics of infection, which require a certain experience [86,87,89]. However, there are on-going optimizations of FDG PET, including utilization of dedicated PET systems and usage of appropriate diagnostic criteria [29,88]. Currently FDG PET re-gains its attraction as a diagnostic tool for peri-prosthetic infections [88].

**Figure 3:**
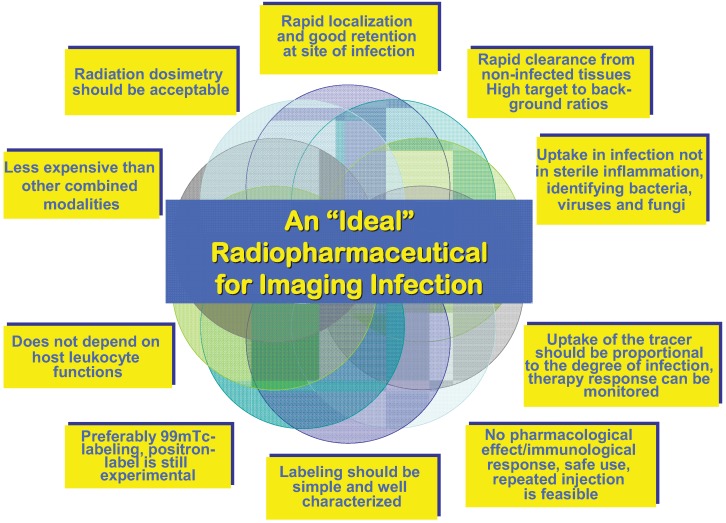
Criteria of an ideal radiopharmaceutical.

In addition to the above mentioned drawbacks of conventional probes to image infection, low grade and hidden infections (bacteria in biofilms and intracellular) are not reached by clinical imaging so far. There is also no quantitative imaging of bacterial burden and viability, which may enable monitoring of antimicrobial therapies. Drug-resistant bacteria are not specifically addressed in conventional diagnostic. Therefore “Search of the Grail” [69], for an ideal infection imaging probe is on-going. The criteria of an “ideal” radiopharmaceutical as defined by [51] are presented in Figure 3. 

In a perspective view, I suppose that the “Grail” search may go into the direction of multiplexed probes. Thus, to assess intracellular bacteria, there shall appear chemically fused bacteria-specific probes, which would contain a fragment enabling intracellular penetration to target bacteria inside eukaryotic cells. Quantitatively bacteria could be assessed by a careful validation of each probe in preclinical settings with a gradual bacterial burden. Viability of bacteria could be assessed by employing probes specific to bacteria permeability. On the other hand, other than radioactivity based imaging modalities such as functional CT and MRI with appropriate contrast agents conjugated to infection probes may arise as an alternative to Nuclear Imaging. 

## 6. Conclusions

Though implant-associated infections occur at low rates, they are a serious health threat for patients, and their clinical management is expensive. Diagnostic of these infections is hampered by bacteria going intracellular, formation of biofilms, aseptic and posttraumatic changes. Intracellular bacteria and bacteria in biofilms are still undetectable. Post-traumatic changes and aseptic processes have similar presentations as infections. Therefore, there is a clinical niche for improved diagnostic tools to uncover pathogenic forms of infections. Imaging technologies hold the promise to become indispensable tools for infection diagnostic and eradication. Nuclear medicine using radiolabeled infection tracers is widely employed for implant-associated infection diagnostic in clinics. Development of multiplexed imaging modalities together with identification of specific infection probes is progressing rapidly. In the next few years new achievements in diagnostic imaging will significantly decrease the risk of late and falsely diagnosed implant-associated infections. 

## Supplementary Files

Supplementary File 1
