# Supplementary material for: Functional Imaging in Diagnostic of Orthopedic Implant-Associated Infections
Source: Diagnostics (Basel). 2013 Oct 21;3(4):356–71. doi: 10.3390/diagnostics3040356 (PMC4665528; doi:10.3390/diagnostics3040356)
Supplement: Supplementary File 1 [file diagnostics-03-00356-s001.docx]

Supplementary Information

**Functional Imaging in Diagnostic of Orthopedic Implant-Associated Infections**

**Appendix 1.** Clinical Diagnostics Tests for Implant-Associated Infections.

| **Test** | **Classification** | **Observations** | **Definition of Positive Result** | **Application** | **Limitations** |
| --- | --- | --- | --- | --- | --- |
| Clinical examination | *Preoperative* | Clinical history  Classical symptoms and signs of infection: severe local pain, fiver, draining periprosthetic sinus | Patients complains and obvious physical abnormalities | - Suggests infection | - Many of the symptoms and signs overlap with those of other clinical conditions such as intra-articular hematoma, instability, aseptic loosening, sterile inflammation etc. |
| X-ray  Anatomical Imaging | *Preoperative* | Inner structure with anatomical details | Visible abnormalities on images | - Primarily used in any diagnosis - X-ray based CT gives high 3D resolution - Multimodality potential: SPECT/CT, PET/CT | - Often, there is no obvious radiographic findings suggestive of bone infection - May show features indistinguishable from those seen in aseptic loosening - Artifacts due to the metal implants |
| Scinti-graphy  Functional Imaging | *Preoperative* | Radioisotopes:  67-Ga, 111-In, 99m-Tc, 18-F *etc*. based probes localize to infection sites | ↑uptake in infected areas | - Radiolabeled WBC is the **“gold standard”** in infection imaging - Combined radiolabeled WBC/bone marrow is the current method of choice for specific infection imaging [1] - 3 phase bone scan: information about time-resolved processes: perfusion to a lesion, relative vascularity, bone-turnover - Multimodality potential: SPECT/CT, PET/CT | - There is no confirmation of “ideal” infection probes/technique to standardize infection imaging - Possible radiation burden, poor biodistribition and clearance - Costs due to sophisticated techniques |
| Serology | *Preoperative* | WBC counts | >11×10^9^/L [2] | - Suggests infection | - Low sensitivity and specificity - Blood handling |
|  |  | Neutrophil percentage in WBC | >75% [2] |  |  |
|  |  | Erythrocyte sedimentation rate, ESR  Normally ERS is ↑ postoperatively and ↓ within 6 weeks | >22.5 mm/hr [3] | - CRP level is more sensitive to infection then ESR - Combined CRP and ESR are very accurate to establish presence or absence of infection prior surgical intervention | - Increased postoperatively in all patients, only delayed diagnosis (3–6 weeks) is possible - Patients who have inflammatory conditions prior implant-related infection also show elevated ESR and CRP levels |
|  |  | C-reactive protein, CRP  Normally CRP level ↑ postoperatively, returns to normal within 3 weeks after an operation | >13.5 mg/L[3] |  |  |
|  |  | Serum Procalcitonin, PCT | >0.5 ng/ml [4] | - Allows early postoperative diagnosis   - Significantly higher levels in infected patients vs non-infected on the days 1-3 after surgery - Higher diagnostic accuracy than ESR/ CRP | - Mechanisms underlying PCT induction during or after surgery have to be elucidated |
|  |  | Interleukin-6, IL-6  IL-6 ↑level returns to normal within 48 after the operation | >10 mg/L[5] | - It is unlikely to be ↑ in patients with aseptic loosening | - Maybe elevated in patients with un underlying inflammatory arthropathy |

| Microbio  logical | *Intraoperative* | Frozen Sections (Histology)  from periprosthetic tissues | >5 neutrophils (or less) in 3 of 400× high power microscopic field [6] | - Intraoperatively confirms an infection when preoperative septic loosening is suspected | - Does not identify causative organism - Related to the experience of the pathologist who interprets the sections - Related to the sampling methods of the surgeon - Has high rates of false-negative results when the infection is due to low-virulence microorganisms |
| --- | --- | --- | --- | --- | --- |
|  |  | - Tissues dissected periprosthetically - Swabs from implant material | > 1/3 of cultures are positive for growing of microorganisms | - The **gold standard** of infection diagnosis | - May fail due to contaminations during probes uptake and transport |
|  |  | Culture of aspirated joint (synovial) fluid | ≥1 growing culture[2] | - Good guess of potential periprosthetic infection | - Appropriate needle placement requires radiologic confirmation - Affected by antiobiotic therapy-best performance 2 weeks after the last antibiotic dose |
|  | *Preoperative/ Intraoperative* | Gram stain of biopsies and fluids | Gram+ bacteria stain pink (crystal violet), Gram- red (safranin) | - Routinely used test - Performed pre and intraoperatively | - Has poor sensitivity and specificity because of widespread preoperative antibiotic therapy |
|  |  | WBC count  Neutrophil percentage | 500/uL[6]  >65% [7] | - Help to distinguish among osteoarthritis, infection and noninfectious inflammatory arthropathies | - Disparity in reported WBC counts |

| Molecular techniques | *Intraoperative* | 16S rRNA PCR | PCR amplification of bacteria DNAl [8] | - Bioflim associated bacteria detection - Bacteria isotypes can be detected, i.e. resistant strains | - Very sensitive to contaminations - Difficult to sample pathogens from unidentified sites of infection - Contaminations of non-pathogenic bacteria - Cannot distinguish between viable and necrotic bacteria - Commercial PCR reagents may include intrinsic bacterial contaminations |
| --- | --- | --- | --- | --- | --- |
|  |  | Microarray | Bacterial mRNA identification | - Numerous mRNA in a single test - Detect only viable bacteria | - Is not widely used in clinics so far - Very expensive |
|  |  | Proteomics | Specific bacterial proteins identification | - Simultaneous isolation and identification of numerous bacteria specific proteins |  |
| **New Methods** Sonication [9] | *Intraoperative* | Sonication-fluid culture | >50 bacteria/mL | - Is more sensitive than just tissue culture - Simple and available technique - Yielded viable microorganisms can be subjected to antimicrobial susceptibility test - Improved detection of polymicrobial infection - Distinguish bw infected (bacteria attached to a prosthesis and removed by sonication) from contaminated during the processing prosthesis | - Lack of gold standard for infection definition - Long processing of explanted components - Do not detect mycobacteria and fungi - Even 2 weeks after a post antimicrobial therapy do not result appropriate culture sensitivity |
| Calorimetry  [10-12] | *Intraoperative* | Viable bacteria generate heat, which can be measured by an isothermal calorimeter producing bacterial specie-specific, heat power-time curves | Heat flow >10 mW above the lowest value of a standard power-time curve | - Rapid (4–10 h) and accurate bacteria detection | - Evaluated in the lab oratory for some bacterial strains and rat models, not widely available in clinics - Multistrain infections have complicated heat power-time curves , which are difficult to interpret |

**Appendix 2.** Infection Imaging Techniques.

| **Technique** | **Working Wave Length, nm** | **Modality** | **Powered by** | **Mechanism** | **Usefulness for Implant Related Imaging** | **Limitations** |
| --- | --- | --- | --- | --- | --- | --- |
| **USG** | >10^9^ | Anatomical | *Contrast agent*  *i.e.* microbubbles | - An acoustic sensor sends pulses of sound into a material. Whenever a sound wave encounters a material with a different density (acoustical impedance), part of the sound wave is reflected back to the sensor and is detected as an echo | - Non-invasive, operator dependent evaluation of musculoskeletal infection - It shows the structure of organs - It has no known long-term side effects and rarely causes any discomfort to the patient - Equipment is widely available and comparatively flexible | - Detection is limited to the soft tissue abnormalities around a bone because the sonic beam cannot cross a bone cortex and identify a bone marrow discontinuity - An early postsurgical diagnosis cannot consistently separate abscess from normal postoperative changes |
| **MRI** | 10^^5^–10^^8^ | Anatomical/  Functional | *Contrast agent*  Gd complexes,  Iron oxide particles | - A powerful rotating magnetic field is used to align a nuclear magnetization of hydrogen atoms in a dielectric surrounding and produce a rotating magnetic field detectable by a scanner - A signal of the rotating field can be manipulated by additional magnetic fields to build up enough information and construct an image of a body | - It has greater contrast and higher anatomical resolution than X-ray and CT - Does not involve ionizing radiation exposure - Useful to detect and determinate the extent of infection - Multimodal (hybrid) imaging using multifunctional probes is possible - Iron oxide particles conjugated to fluorescent dyes or radioisotopes for hybrid MRI/fluorescence or MRI/SPECT, respectively | - Metal implants and prosthetic joints introduce artifacts - An early postsurgical diagnosis cannot consistently separate abscess from normal postoperative changes - High operating costs |
| **X-RAY** | 0.01–10 | Anatomical | *Contrast agent*  Iodinated compounds,  Barium sulfate | - X-ray pulses illuminate a body or limb, with radiographic film placed behind it. Bones that are present absorb most of X-ray photons, because they have a higher electron density than soft tissues. On a developed film soft tissues appear dark and bones - white | - It is the first test in diagnostics of orthopedic pathologies | - Planar imaging - X-rays are inconclusive, non-specific and sometimes misleading - Early infection, until bone or joint severe destruction occur, is not detectable - X-ray is an ionizing irradiation, can induce tumors |
| **CT** | 0.01–10 | Anatomical | *Contrast agent*  Iodine, Barium,  Barium sulfate  Osmium tetraoxide  Gastrografin | - CT is a digital geometry process of a large series of two-dimensional X-ray images taken around a single axis of rotation to generate a three-dimensional image of the inside of an object | - Provides the excellent assessment of bone and soft tissue structures - Cross-sectional images are created with the benefit of high density, contrast and spatial resolution - µCT – improved resolution up to 300 µM - Very low radiation dose | - Metal implants and prosthetic joints introduce artifacts - The early postsurgical diagnosis cannot consistently separate abscess from normal postoperative changes - CT X-ray is an ionizing irradiation, can induce tumors |
| **SCINTIGRAPHY** | <0.01 | Functional | *Radioactive tracer*  (gamma emitters)  99m Tc  111 In  67 Ga | - Radiopharmaceuticals based imaging: radioactive isotopes (99mTc, 111In etc) attached to infection probes are injected into a body. Radioisotopes emit γ-rays, which are detected by γ-cameras. Images are reconstructed from the γ emission patterns. Radioisotopes differ in their half-life; therefore the imaging time has to be adjusted to a particular isotope - 99mTc is the most popular isotope, have the half-life time 6 hours, optimal physical characteristics for γ-camera imaging, biodistribution and body clearance | - Traces musculoskeletal abnormalities and pathologies on the basis of physicochemical changes - Provides the evaluation of bone pathology | - Planar imaging with poor spatial resolution - A radioisotope burden, conjugation chemistry to probes and half-life time are factors of careful balance and potential risk - There is a blood handling related risk when radiolabeled leukocytes are used as infection probes - There is a continuous search for new infection-specific probes, so far none has 100 % specificity to infection in clinical settings |
| **SPECT** | <0.0.1 | Functional | *Radioactive tracer*  (gamma emitters)  99m Tc  111 In  67 Ga | - SPECT is the 3D upgrade of Scintigraphy - Imaging is performed by using a γ-camera to acquire multiple 2-D images (projections), from multiple angles. Then, a computer guided tomography yields a 3-D dataset - Golden standard – 99mTc-WBC (white blood cells) - Method of choice: combined 111 In – WBC/ 99m Tc – bone marrow probe | - 3D spatial resolution - Abnormalities and pathologies recognition similar to Scintigraphy - Combined with CT gives anatomical localization of infection | - If combined with CT – artifacts due to metal implants can appear |
| **PET** | <0.01 | Functional | *Radioactive tracer* (positron emitters)  18 F  68 Ga; 64 Cu; 124 I; 125 I | - Detects γ-rays emitted indirectly by a positron-emitting radionuclide, which is introduced into the body on a biologically active molecule - Most studied probe is 18 F - Fluorodeoxyglucose (18 F – FDG) - A tracer distribution within a body in 3 or 4-dimentional space (4^th^ dimension is time) is digitally reconstructed - Often combined with CT | - Improved imaging quality with respect to Scintigraphy and SPECT - Combined with CT gives anatomical localization of the tracer distribution | - The same as SPECT - Sophisticated instrumentation and high costs - need of a cyclotron and a chemical staff in the vicinity of the clinic - Positron-emitting probes are extremely unstable - 18F-FDG uptake is non-specific to infection |
| **FLUORESCENCE IMAGING** | UV 20–390  Vis 39–780  IR 780–1000 | Functional | *Fluorescent compound*  (photon emitters)  Dyes,  Quantum dots,  Fluorescing proteins | - Fluorophors (dyes, Quantum Dots QD, fluorescing proteins) are attached to specific targets within tissues (or cells). Upon excitation they emit light, which is detected - Genetically encoded fluorescing proteins (GFP-Green Fluorescing Protein, RFP – Red Fluorescing Protein etc) emit light upon excitation (like common organic dyes) - Fused with a protein of interest and transfected into cells they allow studying in vivo behavior of the protein in the cells etc - Wide-field and confocal fluorescence modes differ in the excitation way: parallel and focused (pin-hole) excitations, respectively - In multiphoton two (or more) excitation photons are simultaneously adsorbed by a probe - The excitation light source for a wide-field microscope (WFM) is a fluorescence lamp, while confocal one uses a continuous wave laser and multiphoton one uses a pulsed laser - Bioluminescence (BL) detects light produced by luciferase enzyme reaction with a substrate (or other BL enzyme -substrate pair); no external excitation needed | - Fluorescence techniques enable 3D and *in vivo* imaging: intraoperative and intravital imaging - WFM is an established method for immunohistology tissues and cells - Confocal mode have a improved spatial and 3D resolution - Multiphoton provides a long-wave excitation with less damage and deeper tissue penetration - BL is a good validation tool for *in vivo* small animal infection models | - Non-specific tissue autofluorescence - Poor spatial resolution and poor anatomical resolution of inner structures in WFM and BL - Heat and warm damage of biosamples, poor tissue penetration; dye’s bleaching upon prolonged excitations in WFM and confocal - Possible toxicological burden and slow clearance of dye-based probes (also QD) - Applicable for *ex vivo* and *in vivo* small animal models but has less potential for *in vivo* human clinical imaging |

**Appendix 3.** Clinically Relevant Radiolabeled Probes for Infection Imaging.

| **Probe** | **Uptake mechanisms** | **Applications - Advantages** | **Limitations- Risks** |
| --- | --- | --- | --- |
| **Nonspecific** *Target Bone* | | | |
| - 99mTc-MDP [13] | - An increased vascular permeability and bone metabolism - MDP is involved in the bone turnover and has increased uptake in tumors, fractures and infections | - Bone MDP imaging is widely used as a screening method for skeletal lesions due to low costs and high availability - Static and dynamic bone imaging bw 2 and 24 h - Three-phase bone imaging - combines dynamic and static bone imaging resolved in time - Combined with 67Ga or rWBC is more effective | - Alone does not differentiate bw tumors, aseptic fractures and infections |
| - 18F-NaF [14] | - Involved in bone perfusion and turnover - It is a bone-seeking radiotracer, which adsorbs into the bone crystalline structure of hydroxyapatite (bone matrix) - No protein binding in blood flow - Transported by Red Blood Cells | - Used in clinics - Dynamic 18F-PET provides quantitative estimate of bone metabolism | - Non-specific for infection |
| - 67Ga-citrate [13] - 68Ga-citrate and 68Ga-transferrin [15-17] | - An increased blood flow and vascular permeability - Transported by leukocytes - Ga binds to bacterial siderophores - Ga binds to transferrin, which is overexpressed in inflammatory foci - Ga-transferrin complex at an infection site re-associate into Ga-lactoferrin | - Ga accumulates in both septic and aseptic inflammation, in the bone marrow and in areas of increased bone mineral turnover - Often used in combined with MDP - 67Ga - Used in clinics since 1971 - Imaging for longer period - 68 Ga-citrate - Accurately detect infection within 60 min - Applicable for surgical planning, antibiotics monitoring - Distinguish prosthetic infection from loosening - 68Ga-transferrin - Capable detecting Gram-positive and Gram-negative bacteria | - 67Ga - Low resolution related to high energy emitted by γ-photons - Long physical half-life time, requiring low injection activity due to irradiation concerns - High background activity - 68Ga - Short half-time - very quick imaging period |
| - 99mTc-NC [1,18] | - A nanocolloid (NC) accumulates in the bone marrow due to an increased vascular permeability - Uptake by activated endothelial cells and leukocytes | - Bone marrow imaging - Rapid localization to infection sites within 30- 60 min - Time resolved imaging is possible - Nanocolloids in use are - Stannous fluorid colloid – 1 -3 um cheap WBC labeling - Albumin nanocolloid– Nanocoll ® - 80 nm: blood pool and lymphatic's imaging - Sulfur nanocolloid–NanoCIS–100 nm: bone marrow imaging - Used in combination with 111In-WBC for higher sensitivity in musculoskeletal infections | - Do not image bone periphery - Stannous fluorid and albumin are non-specific for infection and have unfavorable biodistribution - Sulfur nanocolloid should be freshly prepared and used within 2 hours to decrease background |
| - Target Infection Site | | | |
| - 99mTc- and 111In- HIG [13,19] | - Human Immunoglobulin (HIG) accumulation is due to increased vascular permeability and pathogens’ antigen binding | - Screening test for prosthesis infection - Derived from human antibodies, it negates HAMA (human anti-mouse antibodies) | - Non-specific for infection - Unfavorable biodistribution – high physiological uptake in liver, spleen and kidney - Delayed imaging due to delayed blood pool clearance |
| - 111In, 99mTc liposomes [20] | - Extravasation due to increased vascular permeability - Leukocytes (macrophages, also called phagocytes) at an infection site phagocytize the liposomes, trapping them within the infection area | - Imaging in 4 h and re-imaging in 24 h - Can be used as a drug carrier - Stealth ® liposomes: - PEGylated phospholipid bilayer reduces the recognition of the liposome by phagocytes and increases their circulation half-life - PEGylated liposomes are labeled internally with 111In-oxine or 99mTc-HMPAO, 99mTc-HYNIC - Biodistribution and abscess accumulation of Stealth are better represented by 99mTc then 111In | - Non-specific for infection - Not applicable for patients with endocarditis and decreased infusion of liposomes |
| - Avidin-biotin [21] - 2 step - (strept)avidin - 2) 111In-biotin | - Increased vascular permeability and antigen binding - streptavidin localizes nonspecifically to infection sites due to hyperemia and increased vascular permeability; - 2) radiolabeled biotin binds to the extravasated streptavidin | - Easy, low cost, non-toxic | - Non-specific uptake of streptavidin - Development of human-anti-streptavidin antibodies |
| - 1step [22] - 111In-biotin only | - Biotin is a growth factor for human cells and bacteria. In an on-going infection, biotin uptake is elevated due to increased bacterial proliferation rate | - 111In-biotin accurately detects infections - Can be measured 10 min after i.v. biotin injection | - Can be used >25 days after surgery to eliminate trauma related biotin-nonspecific uptake - Biotin is not essential for fungi |
| - 18F-FDG [23] | - Increased glucose uptake by activated leukocytes | - Superior PET imaging characteristics, high target-to-background ratio - Fast 2 - 4 h, combined with CT for anatomical resolution - Low bone marrow/bone uptake - Quantity analysis | - Does not differentiate infection from aseptic loosening - Labeling is not stable, positron-emitting tracers has to be used as prepared due to a short half-life time - High PET operating costs – poor PET availability - Not useful in leucopenia |
| - 99mTc-HPβCD Nanoprobe [24] | - Radiolabeled Nanoparticles of HPβCD – hydroxypropyl-β-cyclodextrin, oligosaccharide derivative interacts with bacterial maltose binding protein | - Distinguish between aseptic loosening and infection | - In a preclinical study - rat |

| - Specific Target White Blood Cells (WBC) | | | |
| --- | --- | --- | --- |
| - 99mTc-HMPAO WBC - 111In-oxine WBC - 18F-FDG/WBC[25] | - Migration of activated leukocytes to infection sites | - Gold Standard for diagnosis of bone infections secondary to trauma and fractures in the study of prosthetic implants - Dual-isotope combined probe 111InWBC/99mTc-NC has the highest clinical imaging accuracy - Noninfectious conditions such as heterotopic ossification, metastatic disease, degenerative arthritis etc do not accumulate WBC - Multiple time points of imaging resolve an early WBC uptake in bone marrow and a later uptake in infection - PET derivatives are available (18F-FDG/WBC) | - Blood handling is hazardous to personal and patients due to probability of HIV and hepatitis infection - The WBC collection and labeling requires 3- 4 hours - Patients under chemotherapy have altered functions of WBC, thus an altered WBC behavior - Not useful in pancytopenia: no sufficient WBC amount - Immune-compromised in diabetes, glucocorticoid medications and HIV infection, which affect WBC function and localization - Partially treated infections (ie antibiotics therapy) may decrease signaling cues for WBC localization - Most used 99mTc-HMPAO partially disassociate from WBC in the bloodstream, and is excreted through kidney (within minutes) and gallbladder (within hours) - The in vitro radio-labeling degrades WBC to an unknown degree |
| - 99mTc- granulocyte mAB [26-30] | - Increased vascular permeability and migration of labeled granulocytes to infection sites | - In vivo labeling of granulocyte population of WBC - No blood handling: no risk of an infection or cross contamination - High accuracy - Explored antibodies: - Anti-NCA-95 IgG, Anti-CD66 lab explored - Anti-NCA-90 Fab’ Leukoscan - Sulesomab ® - 99mTc-sulesomab combined with 99mTc-NC - 99mTc-Fanolesomab (NeutroSpec®) Anti –CD15 - 99mTc - Besilesomab (Scintimun®) - murine IgG antibody | - None of explored antibodies were infection specific - Lung accumulation and circulation clearance delay occurred - HAMA – Human AntiMurine antibody Response - Risk-benefit analysis for clinical use is low - 99mTc-Fanolesomab (NeutroSpec®) Anti –CD15 - Intact murine IgM mAb, was withdrawn from the US market in 2005 due to safety issues |
| - Radiolabeled Interleukin-8 - 99mTc-IL-8 [31-33] | - IL-8 binds with high affinity to receptors expressed on activated neutrophils | - In vivo labeling is possible - High specific activity - Localize infection in 4 - 24 hr - No risk of infection or cross contamination - Rapid clearance from blood and non-target tissues - No uptake in patients with tumors | - Lack of clinical validation - Less useful in neutropenic, non-neutrophil-mediated, low-grade infections - Possible side effects - No differentiation from fungi infections |
| - Target Bacteria | | | |
| - Radiolabeled Antibiotics [34,35] | - Antibiotics target bacterial cell wall, DNA, RNA and protein synthesis - In particular, ciprofloxacin binds to DNA-gyrase enzyme in living bacteria | - 99mTc-Ciprofloxacin – the most studied antibiotics as an infection agent - Commercial names of 99m Tc-Ciprofloxacin are Infecton; Draximage ® - Specific quick bacterial localization 4–24 hr - Low bone marrow uptake - Lack of side effects - Bacteria resistant to Ciprofloxacin still can take up 99mTc-Ciprofloxacin - PET derivatives are available - Also available: 99mTc- Sparfloxacin, Enrofloxacin, Ceftizoxime, Ceftriaxone, Fluconazole, Alafosfalin etc | - For the most studied Ciprofloxacin - Controversial clinical evaluations - Uptake by Neutrophils and activated macrophages - No differentiation bw fungi and bacteria infection - Tc-Ciprofloxacin instability and non-uniform preparation; Tc-Ciprofloxacin bacterial uptake differs then Ciprofloxacin alone - Non-specific binding to bacteria (wash-in/out) |
| - Radiolabeled synthetic  AMP[13,36-38] | - Positively charged antimicrobial peptides (AMP) bind to negatively charged microbial surfaces | - 99mTc-UBI 29-41 synthetic peptide from human ubiquicidin – the most promising infection agent - Discriminate between infections (various bacteria and fungi) and sterile inflammations - Low affinity to host cells - Visualization in 30 min – 2 hr - Monitoring of antimicrobial therapy - Applicable to leukopenic patients - Low probability of bacterial resistance - Favorable biodistibution and clearance - Well tolerated and lack immunological side effects - Easy kit formulation, can be prepared in large amounts - PET derivatives are available | - Lack of clinical validation - No quantitative estimation of infection - No specific discrimination between bacterial strains - Not able to determinate intracellular infection - Bacteriolysis - Low target-to-background ratio |
| - Natural antimicrobial peptides [39-42] and their synthetic analogs [43,44]. Virtually - 1) Nisin - 2) Polymixins - 3) Lysostaphin [45] - 4) RTA3 derived from Streptococcus mitis [46] - 5) Ceragenin CSA-13, a cationic steroid [47] | - Target bacterial surface | - Specific infection recognition and reduced bacterial resistance - 1) Nontoxic, do not interfere with immunomodulation, cheap fermentation production and easy chemical modification, resistant to proteolysis, distinguish against Gram-positive and Gram-negative - 2) Active against Gram-negative, highly active against LPS; Inert to Gram-positive and yeasts, not toxic at low doses - 3) Active against S. aureus, image bacteria in blood flow - 4) Active against Gram-negative, low salt sensitivity, low toxic to mammals - 5) Active against S.aureus and P. aeruginosa | - Weak antimicrobial activity; nonspecific toxicity; susceptibility to proteolysis: weakly target bacteria - 1) To be established in vivo - 2) Nephro and neurotoxic at high doses - 3) To be validated in vivo - 4) To be established in vivo - 5) To be established in vivo |
| - 99mTc- Bacteriophages [48,49] | - Bacteriophages (phages) viruses attach to specific surface receptors, transfer their genetic material into the host cell for reproduction - Some phages have a natural specificity for bacteria | - Bacterial strain specific imaging: performed in vitro | - The specificity of phages in vivo failed so far - Bind both living and heat-killed bacteria - Non-specifically diffuse across endothelial lining - Being viruses, tend to swap genes with each other and other organisms with which they come into contact – risk of genes intercontamination - Clinicians has doubts in viruses as therapeutic or diagnostics agents |
| - Radiolabeled Tymidin Kinase FIAU - rFIAU [50] | - FIAU is a TK substrate, which is phosphorilated and trapped within bacteria | - Efficient accumulation and good retention in infectious foci - Minimal accumulation in non-target organs - No toxicity - Potential for early diagnosis - In human max signal-to –noise ratio reached within 2h - 125I – SPECT and 124I – PET available - Low cost, simple low-hazard preparation, low radiation burden | - Lack of clinical validation - No data on sensitivity/specificity available to date |
| - Immunoglobulins (IG) to Surface-Associated Biofilm Immunogens [51] | - Bind specific bacterial surface-associated proteins, unregulated upon biofilm maturation | - Visualization of bacteria in biofilms - Polyclonal IG can distinguish between gram positive and gram negative; S. aureus and S. epidermidis in biofilms | - Tested in vitro but not in vivo - Antibodies were raised in rabbits and might be not applicable for human - Non-specific to infection |
| - Synthetic inorganic  complexes [52-54] | - Bind anionic surface of bacterial cell wall | - Synthetic zinc (II) –(2,2’-dipicolylamine) complexes target anionic phospholipids of bacteria - Selectively highly active against gram-positive S. aureus - Non-toxic to mammalian cell | - Clinical potential has not been validated |
| - Bacteriophage enzymes “lysine” [55] | - Degrades bacterial cell wall to allow phage release | - Exogenously added lysine can lyse Gram Positive bacteria cell wall, which lead to the bacteria death - “Lysine” enzymes can be used as spray, lozenge, mouthwash, suppository, inhaler, bandages and eye drops - Streptococcal bacteriophage C1 lysin effectively kills streptococci and do not harm mucosal bacteria in mice - S.pneumoniae bacteriophage enzymes Pal and Cpl-1 eliminate targeted bacteria and do not harm human cells and harmless bacteria | - To be established |
| - 68Ga-siderophores [56] | - Siderophores–low-molecular-weight iron chelating molecules produced by bacteria and fungi - 68Ga chelates accumulate specifically in microorganisms | - 68Ga derivative are easy to prepare - Species specific | - Tested in mice |
| - More probes, including Peptides, Cytokines, Chemokines, Interferons, Growth Factors etc can be found in [19,57] | | | |

**Glossary/Abbreviations**

AMP: Antimicrobial peptide

BW: between

FDG: Fluorodeoxyglucose

FIAU: 1-(2’-deoxy-2’-fluoro-β-D-rabinofuranosyl)-5-iodouracil

HAMA: Human Antimurine Response

HIG: Human Immunoglobulin

HMPAO: Hexamethylpropyleneamine Oxime

HYNIC: 6-Hydrazinopyridine-3-Carboxylic Acid chelaor

*i.v.*: intra venous

IL: Interleukins

Liposomes: Microscopic sphere consisting of one or more lipid bilayers surrounding an aqueous-filled space

mAB: Monoclonal Antibody

MDP: Methylene disphosphonate

NC: Nanocolloid

NHS-MEG3: N-Hydroxysuccinimide ester of mercaptoacetyltriglycine chelator

TK: Thymidine Kinase

WBC: White Blood Cells

UBI29-41: Ubiquicidin fragment 29-41

**References**

1. Palestro, C. J.; Love,C.; Tronco,G.G. et al. Combined labeled leukocyte and technetium 99m sulfur colloid bone marrow imaging for diagnosing musculoskeletal infection. *Radiographics* **2006**, 26, 859-870.

2. Spangehl, M. J.; Masri,B.A.; O'Connell,J.X. et al. Prospective analysis of preoperative and intraoperative investigations for the diagnosis of infection at the sites of two hundred and two revision total hip arthroplasties. *J.Bone Joint Surg.Am.* **1999**, 81, 672-683.

3. Greidanus, N. V.; Masri,B.A.; Garbuz,D.S. et al. Use of erythrocyte sedimentation rate and C-reactive protein level to diagnose infection before revision total knee arthroplasty. A prospective evaluation. *J.Bone Joint Surg.Am.* **2007**, 89, 1409-1416.

4. Hunziker, S.; Hugle,T.; Schuchardt,K. et al. The value of serum procalcitonin level for differentiation of infectious from noninfectious causes of fever after orthopaedic surgery. *J.Bone Joint Surg.Am.* **2010**, 92, 138-148.

5. Di Cesare, P. E.; Chang,E.; Preston,C.F. et al. Serum interleukin-6 as a marker of periprosthetic infection following total hip and knee arthroplasty. *J.Bone Joint Surg.Am.* **2005**, 87, 1921-1927.

6. Bauer, T. W.; Parvizi,J.; Kobayashi,N. et al. Diagnosis of periprosthetic infection. *J.Bone Joint Surg.Am.* **2006**, 88, 869-882.

7. Trampuz, A.; Hanssen,A.D.; Osmon,D.R. et al. Synovial fluid leukocyte count and differential for the diagnosis of prosthetic knee infection. *Am.J.Med.* **2004**, 117, 556-562.

8. Tunney, M. M.; Patrick,S.; Curran,M.D. et al. Detection of prosthetic hip infection at revision arthroplasty by immunofluorescence microscopy and PCR amplification of the bacterial 16S rRNA gene. *J.Clin.Microbiol.* **1999**, 37, 3281-3290.

9. Trampuz, A.; Piper,K.E.; Hanssen,A.D. et al. Sonication of explanted prosthetic components in bags for diagnosis of prosthetic joint infection is associated with risk of contamination. *J.Clin.Microbiol.* **2006**, 44, 628-631.

10. Trampuz, A.; Steinhuber,A.; Wittwer,M. et al. Rapid diagnosis of experimental meningitis by bacterial heat production in cerebrospinal fluid. *BMC.Infect.Dis.* **2007**, 7, 116-

11. Trampuz, A.; Salzmann,S.; Antheaume,J. et al. Microcalorimetry: a novel method for detection of microbial contamination in platelet products. *Transfusion* **2007**, 47, 1643-1650.

12. Baldoni, D.; Hermann,H.; Frei,R. et al. Performance of microcalorimetry for early detection of methicillin resistance in clinical isolates of Staphylococcus aureus. *J.Clin.Microbiol.* **2009**, 47, 774-776.

13. Gemmel, F.; Dumarey,N.; Welling,M. Future diagnostic agents. *Semin.Nucl.Med.* **2009**, 39, 11-26.

14. Wong, K. K.; Piert,M. Dynamic Bone Imaging with 99mTc-Labeled Diphosphonates and 18F-NaF: Mechanisms and Applications. *J.Nucl.Med.* **2013**, 54, 590-599.

15. Kumar, V.; Boddeti,D.K. (68)Ga-radiopharmaceuticals for PET imaging of infection and inflammation. *Recent Results Cancer Res* **2013**, 194, 189-219.

16. Kumar, V.; Boddeti,D.K.; Evans,S.G. et al. (68)Ga-Citrate-PET for diagnostic imaging of infection in rats and for intra-abdominal infection in a patient. *Curr.Radiopharm.* **2012**, 5, 71-75.

17. Kumar, V.; Boddeti,D.K.; Evans,S.G. et al. Potential use of 68Ga-apo-transferrin as a PET imaging agent for detecting Staphylococcus aureus infection. *Nucl.Med.Biol.* **2011**, 38, 393-398.

18. Palestro, C. J.; Mehta,H.H.; Patel,M. et al. Marrow versus infection in the Charcot joint: indium-111 leukocyte and technetium-99m sulfur colloid scintigraphy. *J.Nucl.Med.* **1998**, 39, 346-350.

19. Signore, A.; Mather,S.J.; Piaggio,G. et al. Molecular imaging of inflammation/infection: nuclear medicine and optical imaging agents and methods. *Chem.Rev.* **2010**, 110, 3112-3145.

20. Oyen, W. J.; Boerman,O.C.; Storm,G. et al. Detecting infection and inflammation with technetium-99m-labeled Stealth liposomes. *J.Nucl.Med.* **1996**, 37, 1392-1397.

21. Lazzeri, E.; Pauwels,E.K.; Erba,P.A. et al. Clinical feasibility of two-step streptavidin/111In-biotin scintigraphy in patients with suspected vertebral osteomyelitis. *Eur.J.Nucl.Med.Mol.Imaging* **2004**, 31, 1505-1511.

22. Lazzeri, E.; Erba,P.; Perri,M. et al. Clinical impact of SPECT/CT with In-111 biotin on the management of patients with suspected spine infection. *Clin.Nucl.Med.* **2010**, 35, 12-17.

23. Glaudemans, A. W.; Signore,A. FDG-PET/CT in infections: the imaging method of choice? *Eur.J.Nucl.Med.Mol.Imaging* **2010**, 37, 1986-1991.

24. Shukla, J.; Arora,G.; Kotwal,P.P. et al. Radiolabeled oligosaccharides nanoprobes for infection imaging. *Hell.J.Nucl.Med.* **2010**, 13, 218-223.

25. Walker, R. C.; Jones-Jackson,L.B.; Martin,W. et al. New imaging tools for the diagnosis of infection. *Future.Microbiol.* **2007**, 2, 527-554.

26. Signore, A.; Prasad,V.; Malviya,G. Monoclonal antibodies for diagnosis and therapy decision making in inflammation/infection. Foreword. *Q.J.Nucl.Med.Mol.Imaging* **2010**, 54, 571-573.

27. Goldsmith, S. J.; Signore,A. An overview of the diagnostic and therapeutic use of monoclonal antibodies in medicine. *Q.J.Nucl.Med.Mol.Imaging* **2010**, 54, 574-581.

28. Love, C.; Palestro,C.J. 99mTc-fanolesomab Palatin Technologies. *IDrugs.* **2003**, 6, 1079-1085.

29. Sousa, R.; Massada,M.; Pereira,A. et al. Diagnostic accuracy of combined 99mTc-sulesomab and 99mTc-nanocolloid bone marrow imaging in detecting prosthetic joint infection. *Nucl.Med.Commun.* **2011**, 32, 834-839.

30. Richter, W. S.; Ivancevic,V.; Meller,J. et al. 99mTc-besilesomab (Scintimun) in peripheral osteomyelitis: comparison with 99mTc-labelled white blood cells. *Eur.J.Nucl.Med.Mol.Imaging* **2011**, 38, 899-910.

31. Gratz, S.; Rennen,H.J.; Boerman,O.C. et al. (99m)Tc-interleukin-8 for imaging acute osteomyelitis. *J.Nucl.Med.* **2001**, 42, 1257-1264.

32. Krause, S.; Rennen,H.J.; Boerman,O.C. et al. Preclinical evaluation of technetium 99m-labeled P1827DS for infection imaging and comparison with technetium 99m IL-8. *Nucl.Med.Biol.* **2007**, 34, 925-932.

33. Bleeker-Rovers, C. P.; Rennen,H.J.; Boerman,O.C. et al. 99mTc-labeled interleukin 8 for the scintigraphic detection of infection and inflammation: first clinical evaluation. *J.Nucl.Med.* **2007**, 48, 337-343.

34. Lambrecht, F. Y. Evaluation of (9)(9)(m)Tc-labeled antibiotics for infection detection. *Ann.Nucl.Med.* **2011**, 25, 1-6.

35. Benitez, A.; Roca,M.; Martin-Comin,J. Labeling of antibiotics for infection diagnosis. *Q.J.Nucl.Med.Mol.Imaging* **2006**, 50, 147-152.

36. Welling, M. M.; Mongera,S.; Lupetti,A. et al. Radiochemical and biological characteristics of 99mTc-UBI 29-41 for imaging of bacterial infections. *Nucl.Med.Biol.* **2002**, 29, 413-422.

37. Ferro-Flores, G.; Ramirez,F.M.; Melendez-Alafort,L. et al. Peptides for in vivo target-specific cancer imaging. *Mini.Rev.Med.Chem.* **2010**, 10, 87-97.

38. Akhtar, M. S.; Imran,M.B.; Nadeem,M.A. et al. Antimicrobial peptides as infection imaging agents: better than radiolabeled antibiotics. *Int.J.Pept.* **2012**, 2012, 965238-965257.

39. Oyston, P. C.; Fox,M.A.; Richards,S.J. et al. Novel peptide therapeutics for treatment of infections. *J.Med.Microbiol.* **2009**, 58, 977-987.

40. Lohner, K. New strategies for novel antibiotics: peptides targeting bacterial cell membranes. *Gen.Physiol Biophys.* **2009**, 28, 105-116.

41. Rathinakumar, R.; Walkenhorst,W.F.; Wimley,W.C. Broad-spectrum antimicrobial peptides by rational combinatorial design and high-throughput screening: the importance of interfacial activity. *J.Am.Chem.Soc.* **2009**, 131, 7609-7617.

42. Sang, Y.; Blecha,F. Antimicrobial peptides and bacteriocins: alternatives to traditional antibiotics. *Anim Health Res.Rev.* **2008**, 9, 227-235.

43. Liu, L.; Xu,K.; Wang,H. et al. Self-assembled cationic peptide nanoparticles as an efficient antimicrobial agent. *Nat.Nanotechnol.* **2009**, 4, 457-463.

44. Vaara, M. New approaches in peptide antibiotics. *Curr.Opin.Pharmacol.* **2009**, 9, 571-576.

45. Potapova, I.; Eglin,D.; Laschke,M.W. et al. Two-step labeling of Staphylococcus aureus with Lysostaphin-Azide and DIBO-Alexa using click chemistry. *J.Microbiol.Methods* **2013**, 92, 90-98.

46. Hawrani, A.; Howe,R.A.; Walsh,T.R. et al. Thermodynamics of RTA3 peptide binding to membranes and consequences for antimicrobial activity. *Biochim.Biophys.Acta* **2010**

47. Bucki, R.; Sostarecz,A.G.; Byfield,F.J. et al. Resistance of the antibacterial agent ceragenin CSA-13 to inactivation by DNA or F-actin and its activity in cystic fibrosis sputum. *J.Antimicrob.Chemother.* **2007**, 60, 535-545.

48. Rusckowski, M.; Gupta,S.; Liu,G. et al. Investigation of four (99m)Tc-labeled bacteriophages for infection-specific imaging. *Nucl.Med.Biol.* **2008**, 35, 433-440.

49. Rusckowski, M.; Gupta,S.; Liu,G. et al. Investigations of a (99m)Tc-labeled bacteriophage as a potential infection-specific imaging agent. *J.Nucl.Med.* **2004**, 45, 1201-1208.

50. Bettegowda, C.; Foss,C.A.; Cheong,I. et al. Imaging bacterial infections with radiolabeled 1-(2'-deoxy-2'-fluoro-beta-D-arabinofuranosyl)-5-iodouracil. *Proc.Natl.Acad.Sci.U.S.A* **2005**, 102, 1145-1150.

51. Brady, R. A.; Leid,J.G.; Kofonow,J. et al. Immunoglobulins to surface-associated biofilm immunogens provide a novel means of visualization of methicillin-resistant Staphylococcus aureus biofilms. *Appl.Environ.Microbiol.* **2007**, 73, 6612-6619.

52. Leevy, W. M.; Lambert,T.N.; Johnson,J.R. et al. Quantum dot probes for bacteria distinguish Escherichia coli mutants and permit in vivo imaging. *Chem.Commun.(Camb.)* **2008**2331-2333.

53. Leevy, W. M.; Gammon,S.T.; Johnson,J.R. et al. Noninvasive optical imaging of staphylococcus aureus bacterial infection in living mice using a Bis-dipicolylamine-Zinc(II) affinity group conjugated to a near-infrared fluorophore. *Bioconjug.Chem.* **2008**, 19, 686-692.

54. DiVittorio, K. M.; Leevy,W.M.; O'Neil,E.J. et al. Zinc(II) coordination complexes as membrane-active fluorescent probes and antibiotics. *Chembiochem.* **2008**, 9, 286-293.

55. Sandeep, K. Bacteriophage precision drug against bacterial infections. *Current Science* **2006**, 90, 631-633.

56. Petrik, M.; Haas,H.; Dobrozemsky,G. et al. 68Ga-siderophores for PET imaging of invasive pulmonary aspergillosis: proof of principle. *J.Nucl.Med.* **2010**, 51, 639-645.

57. Sasser, T. A.; Van Avermaete,A.E.; White,A. et al. Bacterial Infection Probes and Imaging Strategies in Clinical Nuclear Medicine and Preclinical Molecular Imaging. *Curr.Top.Med.Chem.* 2013.
